# Inhibitors of MyD88-Dependent Proinflammatory Cytokine Production Identified Utilizing a Novel RNA Interference Screening Approach

**DOI:** 10.1371/journal.pone.0007029

**Published:** 2009-09-15

**Authors:** John S. Cho, Yun C. Kim, Sherie L. Morrison

**Affiliations:** Department of Microbiology, Immunology and Molecular Genetics, David Geffen School of Medicine, University of California Los Angeles, Los Angeles, California, United States of America; New York University School of Medicine, United States of America

## Abstract

**Background:**

The events required to initiate host defenses against invading pathogens involve complex signaling cascades comprised of numerous adaptor molecules, kinases, and transcriptional elements, ultimately leading to the production of proinflammatory cytokines, such as tumor necrosis factor alpha (TNF-α). How these signaling cascades are regulated, and the proteins and regulatory elements participating are still poorly understood.

**Results:**

We report here the development a completely random short-hairpin RNA (shRNA) library coupled with a novel forward genetic screening strategy to identify inhibitors of Toll-like receptor (TLR) dependent proinflammatory responses. We developed a murine macrophage reporter cell line stably transfected with a construct expressing diphtheria toxin-A (DT-A) under the control of the TNF-α-promoter. Stimulation of the reporter cell line with the TLR ligand lipopolysaccharide (LPS) resulted in DT-A induced cell death, which could be prevented by the addition of an shRNA targeting the TLR adaptor molecule MyD88. Utilizing this cell line, we screened a completely random lentiviral short hairpin RNA (shRNA) library for sequences that inhibited TLR-mediated TNF-α production. Recovery of shRNA sequences from surviving cells led to the identification of unique shRNA sequences that significantly inhibited TLR4-dependent TNF-α gene expression. Furthermore, these shRNA sequences specifically blocked TLR2 but not TLR3-dependent TNF-α production.

**Conclusions:**

Thus, we describe the generation of novel tools to facilitate large-scale forward genetic screens in mammalian cells and the identification of potent shRNA inhibitors of TLR2 and TLR4- dependent proinflammatory responses.

## Introduction

Mammalian Toll-like receptors (TLRs) are single-spanning membrane proteins that display a conserved cytoplasmic Toll−interleukin 1 (IL-1) receptor (TIR) domain motif [1]. Individual TLRs recognize a distinct repertoire of conserved microbial products and are critical mediators of the innate immune response to infection [2]. For example, TLR4 recognizes LPS, an integral cell wall component of Gram-negative bacteria [3], [4], [5], TLR2 recognizes peptidoglycan [6], [7] and bacterial lipoproteins [8], [9], [10], and TLR3 recognizes double stranded RNA (dsRNA), which is produced by many viruses during replication [11].

As a result of shared, cytoplasmic TIR domains, all TLRs utilize similar signaling pathways. In the MyD88-dependent pathway, recruitment of the adaptor protein MyD88 leads to the production of proinflammatory cytokines, such as TNF-α, through the sequential activation of intracellular signaling molecules such as IL-1R-associated kinase (IRAK1) and TNFR-associated factor 6 (TRAF6) [12], [13]. Alternatively, in the MyD88 independent pathway, recruitment of the adaptor molecule TIR domain-containing adaptor-inducing IFN-β (TRIF) can result in the production of type I interferons through the activation of IFN regulatory factor 3 (IRF3) [14]. Signaling through the MyD88-independent pathway can also induce TNF-α production, albeit in a delayed manner, through activation of IRF3 [15]. With the exception of TLR3 and TLR4, all TLRs signal exclusively through the MyD88-dependent pathway. TLR3 is activated solely through the MyD88-independent (TRIF) pathway and is essential for anti-viral responses [14]. TLR4 is unique in that both MyD88-dependent and independent pathways can become activated following its ligation [16], [17].

Full activation of TLR signals is essential for the elimination of invading pathogens [18], [19]. However, tight control of TLR responses is critical as excessive TLR activation can result in immunopathological conditions such as endotoxin shock and chronic autoimmune disease [20]. As many of the mechanisms essential in controlling TLR proinflammatory signals are still unclear, new strategies to identify regulators of TLR signaling are needed.

Short hairpin RNA (shRNA) mediated loss-of-function screens in mammalian cells are powerful tools for the discovery of novel gene functions. To date, two general strategies to develop shRNA libraries have been described. The first requires the synthesis of individual shRNAs targeting each gene of the genome [21], [22]. Alternatively, several groups have generated shRNA libraries from pools of double stranded cDNAs [23], [24], [25], [26]. Both of these strategies have been successfully used in large-scale mammalian screens to identify novel gene functions in various biological processes demonstrating the power of the shRNA library approach [21], [22], [27], [28], [29], [30], [31]. However, these aforementioned RNAi libraries have limited sequence diversity and are restricted to the identification of known protein coding genes or highly expressed cDNA populations. The generation of an RNAi library that is random at the nucleotide level with unrestricted gene perturbation potential could overcome the limitations of traditional RNAi libraries.

We report the generation of a completely random shRNA library and a novel reporter cell line for the efficient identification of shRNAs with the desired phenotype. Using these tools, we performed a large-scale genomic screen to identify shRNA sequences which inhibit LPS-induced TNF-α production.

## Results

### Generation of a random shRNA library

**Figure 1:**
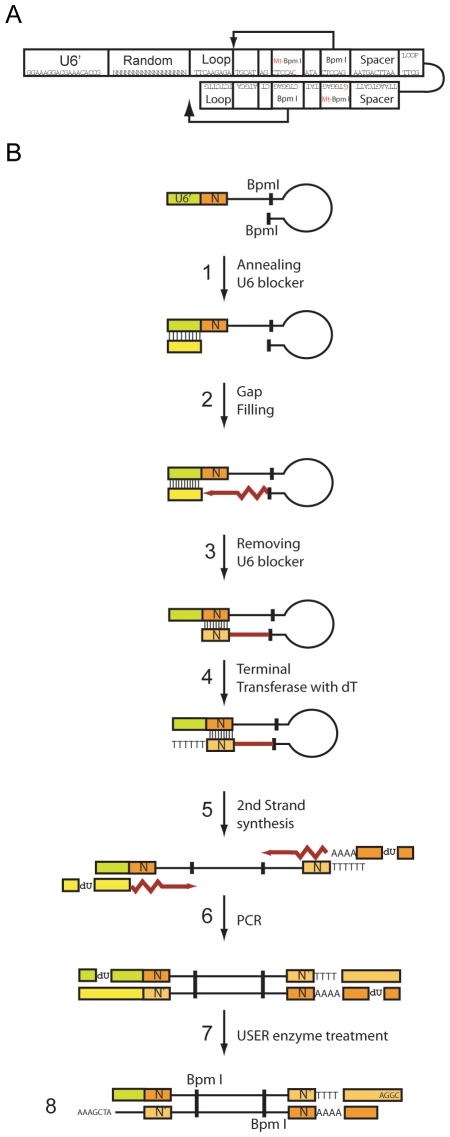
Schematic diagram of random shRNA library construction. (A) Backbone of oligonucleotide used for generation of shRNA library. (B.1–2) First, the 120 bp oligonucleotide containing 20 bp of the 3′ end of U6 including a “G” to initiate transcription, 18 random nucleotides (sense) and a stem-loop structure that can act as a primer for synthesizing the strand complementary to the random 18 bp (anti-sense) was extended using T4 DNA polymerase in the presence of a blocking primer which annealed to the U6 promoter region. (B.3–4) Following purification of the extended oligonucleotide, a poly-thymidine tract was added using terminal transferase (TdT). (B.5) Exo^-^ klenow fragment was used to make the oligonucleotide double stranded using a poly-A oligonucleotide as a primer. (B.6) The purified double stranded DNA was amplified using uracil containing primers. (B.7) The PCR product was digested with USER enzyme to generate overhangs to facilitate cloning. (B.8) The PCR fragment was cloned into the lentiviral vector pLL3.7, and digested with BpmI to remove the extra sequence between the random sense and antisense sequence, leaving a 9 base pair loop sequence.

The procedure for generating a completely random shRNA library is outlined in Figure 1. First, a 120 bp oligonucleotide containing 20 bp of the 3′ end of U6 including a “G” to initiate transcription, 18 random nucleotides (sense), and a stem-loop structure that can act as a primer for synthesizing the strand complementary to the random 18-bp (anti-sense) was made (Fig. 1A). To stop DNA polymerization at the end of the anti-sense 19-bp, a blocking primer containing the sequence complementary to the U6 promoter region was added to anneal to the U6 promoter in the extension reaction (Fig. 1B.1). Since T4 DNA polymerase lacks strand displacement activity, the polymerase will stop transcription upon reaching the 5′ end of the blocking primer (Fig. 1B.2). Following purification of the extended oligonucleotide (Fig. 1B.3), we used terminal transferase (TdT) to attach a poly-thymidine tract, which is recognized as a termination sequence by RNA polymerase III, at the end of the oligonucleotide (Fig. 1B.4). To make the oligonucleotide double stranded, we utilized Exo^-^ klenow fragment, which has strong strand displacement activity and lacks exonuclease activity, and a poly-A oligonucleotide, which can bind to the poly-thymidine tract of the oligonucleotide (Fig. 1B.5). This purified double-stranded DNA was used as a template and amplified using uracil containing primers (Fig. 1B.6). Digestion of the PCR product with USER enzyme (Fig. 1B.7), which digests uracil residues, was used to generate 7 and 4 base pair overhangs to facilitate cloning (Fig. 1B.8). Following cloning into the lentiviral vector pLL3.7, the extra sequence between the random sense and antisense sequence was removed by BpmI digestion and re-ligated, leaving a 9 base pair loop sequence [32]. Importantly, the entire procedure required only one PCR amplification step, minimizing the possibility of sequence specific bias in the generation of the library.

The average transformation efficiency was calculated to be 2×10^8^ colonies per ligation reaction. One hundred duplicate electro-transformations were performed resulting in 2×10^10^ total transformants. As the amount of starting oligonucleotide used was in excess and sufficient to cover all possible random sequences, the diversity of the library was estimated to be 2×10^10^ unique sequences.

### Sequencing results

After the library was constructed, 200 clones were randomly sequenced. Among the 200 clones, 154 had the expected shRNA sequence architecture (i.e., sense19-mer – loop – antisense 19-mer) (data not shown). This result implies that about 77% of the library contains the appropriate shRNA construct, indicating a functional library size of approximately 1.5×10^10^. Among the incorrect constructs, 22% contained sequences that lacked inverted repeats, 6% contained an incorrect loop sequence, and 2% lacked the poly-T transcription termination sequence. Importantly, all 154 correct sequences were unique, confirming the randomness of the shRNA library.

### Generation of TNF-DTA reporter cell line

**Figure 2:**
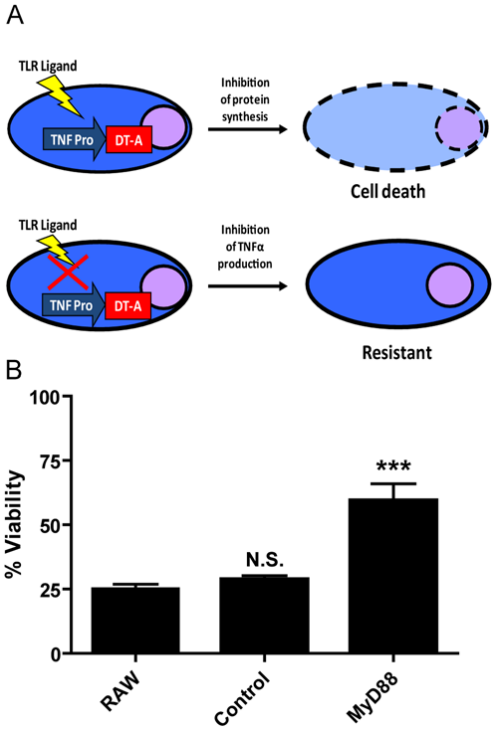
Schematic of macrophage reporter cell line. (A) Schematic of macrophage reporter cell line. The murine macrophage-like cell line RAW 264.7 was stably co-transfected with the TNF-TetO_2_-Tox176 reporter plasmid and the Tet-repressor expression plasmid, PCDNA6/TR. A stable clone was isolated which exhibited the following properties: upon stimulation with lipopolysaccharide and doxycycline, cells expressed DT-A which resulted in cell death (top). However, when transduced with shRNAs which inhibited LPS induced TNF-α production, cells were resistant to LPS induced cytotoxicity (bottom). (B) RAW 264.7 mock infected (RAW), non-specific shRNA (control), and MyD88 specific shRNA infected TNF-Tox cells were stimulated with LPS (2 µg/ml) and doxycycline (1 µg/ml) or just medium alone. Following forty eight hours of stimulation, cell viability was measured using the MTS assay. Percent viability reflects the Abs. 490 of stimulated cells divided by the Abs. 490 of cells treated with medium alone times 100. Data are representative of two independent experiments performed in triplicate. N.S. = not significant, *** = p<0.001.

To identify shRNAs which inhibit LPS induced proinflammatory signals, we generated a murine macrophage reporter cell line (RAW 264.7) that ectopically expresses the diphtheria toxin A (DT-A) fragment driven by the murine TNF-α promoter (Fig. 2A). Thus, cells transduced with an shRNA which inhibits genes necessary for TLR-dependent proinflammatory signals would be positively selected and survive, whereas cells transduced with an irrelevant shRNA would express DT-A and die. Importantly, DT-A cannot enter living cells in the absence of the DT-B fragment [33]. As a result, expression of DT-A alone in cells transduced with an irrelevant shRNA would not have bystander activity to neighboring cells transduced with an shRNA that inhibits TNF-α production (data not shown).

**Figure 3:**
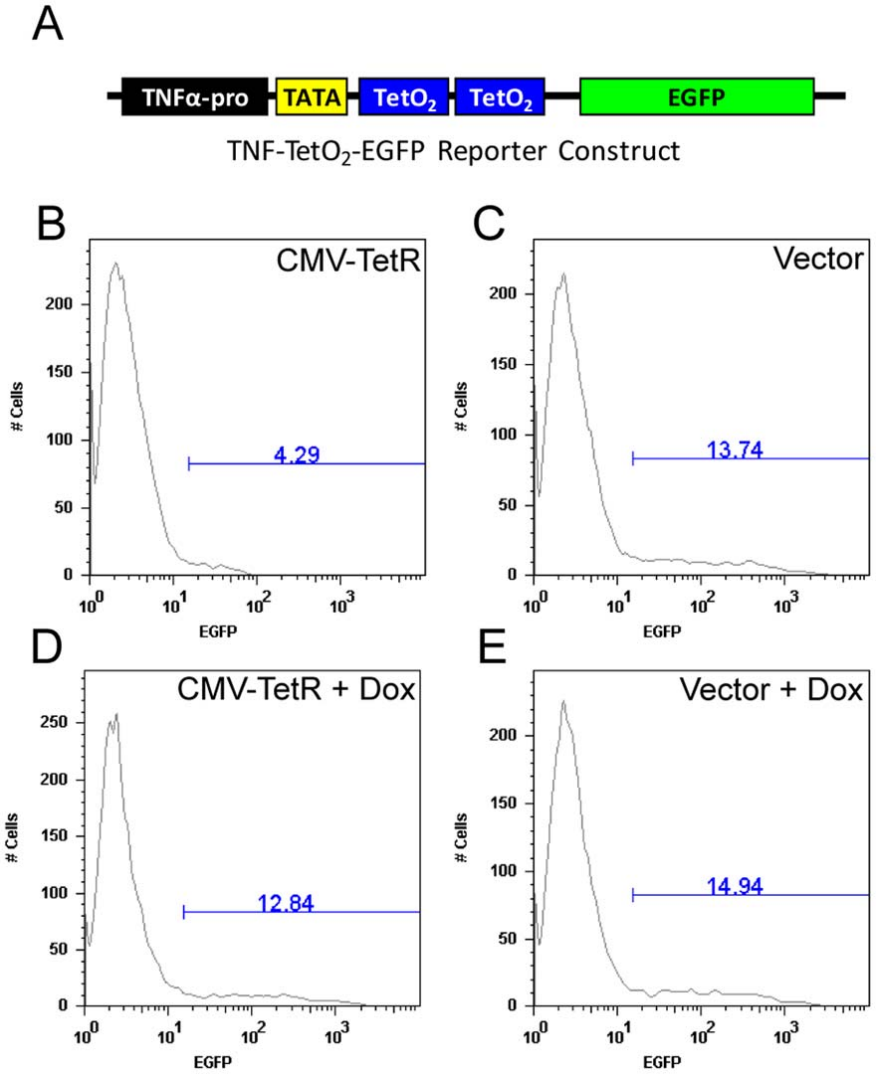
Tet-repressor can reversible inhibit basal TNF-α promoter activity. (A) Schematic diagram of the TNF-TetO_2_-EGFP reporter construct. Two Tet operator binding sites (TetO_2_) were cloned 10 bp downstream of the TNF-α promoter TATA box. (B–E) HEK-293T cells were cotransfected with TNF-TetO_2_-EGFP reporter construct and (B and D) pcDNA6/TR which encodes the Tet-repressor driven by the CMV promoter (CMV-TetR) or (C and E) pcDNA3.1 vector control (Vector) in the presence of doxycycline (DOX) or vehicle. EGFP expression was monitored 48 hours later by flow cytometry.

Due to the toxicity of DT-A, several safeguards were necessary to prevent spontaneous cell death. First, wild type DT-A proved to be too toxic as cells transfected with a TNF alpha reporter plasmid driving wild type DT-A died spontaneously (data not shown). Thus, we used the attenuated form of DT-A (Tox-176) which has been shown to be 10–100 fold less active than the wild type DT-A [34]. Second, the TNF-α promoter exhibits high basal levels of gene expression (Fig. 3C). To obtain tight control of DT-A gene expression, we modified the reporter plasmid to introduce two TetO_2_ operator sites in the TNF-α promoter and used GFP fluorescence as readout for TNF-α promoter activity (Fig. 3A). Cotransfection of a plasmid encoding the Tet-repressor efficiently suppressed the basal level of the TNF-α promoter activity by three-fold (compare Fig. 3B vs. 3C). Importantly, Tet-repressor mediated suppression of TNF-α promoter activity was reversible by the addition of the inhibitor of Tet-repressor, doxycycline (compare Fig. 3B vs. 3D).

Isolation of macrophage clones stably expressing both the attenuated form of DT-A (Tox-176), under the control of a Tet-repressor sensitive TNF-α promoter, and the Tet-repressor (hereafter referred to as TNF-Tox cells) proved to be the most efficient, as these clones were highly sensitive to LPS-induced cytotoxicity and grew *in vitro* with kinetics similar to those of the parental RAW 264.7 cells (data not shown).

To determine if downregulation of a known component of the TLR signaling pathway would inhibit LPS-induced cytotoxicity, we infected TNF-Tox cells with lentivirus expressing an shRNA targeting MyD88, a critical adaptor molecule required for LPS-dependent proinflammatory signals. Indeed, downregulation of MyD88 in TNF-Tox reporter cells resulted in a 3-fold increase in cell viability compared to mock infected cells (Fig. 2B). The expression of a scrambled non-specific control shRNA sequence did not result in an increase in cell viability, indicating that the increased survival of MyD88 shRNA infected cells was a direct result of MyD88 downregulation. Thus, the expression of an shRNA targeting a molecule known to be essential for the induction of LPS induced proinflammatory signals can protect the TNF-Tox reporter cells from LPS-induced cytotoxicity, a proof-of-principle that the reporter cell line is robust.

### Large-scale shRNA screening

The random shRNA library was transduced into the TNF-Tox reporter cell line at a concentration of virus chosen to ensure an average of one copy of shRNA per cell. Seventy two hours post-infection, the cells were stimulated with LPS and doxycycline and monitored for survival. The genomic DNA from surviving cells was harvested and PCR was used to amplify the enriched shRNA sequences. Amplified DNAs were re-cloned into the lentiviral vector and used to make fresh viral stocks containing the enriched shRNA sequences. These sequences were subsequently transduced into fresh TNF-Tox cells and the selection procedure was repeated. Following five rounds of enrichment, we observed a significant increase in cell survival compared to TNF-Tox cells infected with a scrambled non-specific shRNA sequence (data not shown). Again, PCR was used to amplify the shRNA sequences from genomic DNA. The amplified DNAs were cloned into the lentiviral vector and 140 clones were sequenced (Table 1).

**Table 1:** Sequence of 19-mer sense region of recovered shRNAs.

Name	Sense sequence (5′->3′)	Name	Sense sequence (5′->3′)	Name	Sense sequence (5′->3′)
T5-73	CAATCATCAGTGCTCCCCC	**T5-16**	**GAGCTAATCAGCATACAGT**	T5-4	GTAAATATACCTGTCGTAT
T5-76	GAAACGAGGTGCAATTTAC	**T5-44**	**GAGCTAATCAGCATACAGT**	T5-42	GTAAATATTTGAATGTTTA
T5-21	GAAATTATAGATACGGGGT	T5-124	GAGGGAGATGGCAATTTTC	T5-61	GTAACTCGTGGTGCGTATA
T5-86	GAAGTACGGTGGAGTATAG	T5-24	GAGTACTACGTAAGGTATG	T5-101	GTAAGTACGTTCCGTCAAC
T5-72	GAAGTGTCCGTATGTAATG	T5-26	GAGTATTACGTAAGGTATG	T5-121	GTACTAGGATTCGGGGTTC
T5-78	GAAGTTAATATGTGTTTTA	T5-82	GAGTATTACGTCTTCTAGA	**T5-39**	**GTACTGCAGTTGCATACAG**
**T5-17**	**GAATAACGATCAGTTATAG**	T5-100	GATAATTTGGAAGTGCTTA	**T5-74**	**GTACTGCAGTTGCATACAG**
**T5-83**	**GAATAACGGTCAGTTATAG**	**T5-117**	**GATAGATACAGCTCAAAAT**	**T5-92**	**GTACTGCAGTTGCATACAG**
T5-102	GAATACGTCATCAGGGTAG	**T5-133**	**GATAGATACAGCTCAAAAT**	T5-94	GTAGATCGCTCCACCCATG
T5-15	GAATAGAGTGCGTGAAGAC	T5-109	GCATGATTGTCTCCTTGTT	T5-37	GTAGGTAAGTGTCTCAATG
**T5-126**	**GAATATTACGTAAGGTATG**	T5-111	GCCAATTACGTAGTTATGA	**T5-48**	**GTAGTATTGCTATTATGTC**
**T5-5**	**GAATATTACGTAAGGTATG**	T5-62	GATAGTTCCGCTTCTTTCG	**T5-122**	**GTAGTATTGCTATTATGTC**
**T5-9**	**GAATATTACGTAAGGTATG**	T5-96	GATCAGGAGTCACAAAGTC	**T5-134**	**GTAGTATTGCTATTATGTC**
**T5-47**	**GAATATTACGTAAGGTATG**	T5-128	GATCGCGTTCAGGTATTCA	T5-123	GTAGTTGGCACATGTACAG
**T5-49**	**GAATATTACGTAAGGTATG**	T5-104	GATTAGTGACTAGTCATCA	T5-103	GTAGTTTTTTCGCTTCGCG
**T5-52**	**GAATATTACGTAAGGTATG**	**T5-120**	**GATTATCACCCCATGCCGT**	**T5-18**	**GTTAGTTCTGTGTTCTCAG**
**T5-55**	**GAATATTACGTAAGGTATG**	**T5-127**	**GATTATCACCCCATGCCGT**	**T5-81**	**GTTAGTTCTGTGTTCTCAG**
**T5-84**	**GAATATTACGTAAGGTATG**	T5-97	GATTGCAGCCGTAGATTAG	**T5-140**	**GTTAGTTCTGTGTTCTCAG**
**T5-85**	**GAATATTACGTAAGGTATG**	T5-132	GCAAACGGGCTTCGCAAGA	T5-22	GTCATTTTCGTGCTCAGCA
T5-99	GAATCTCAGTATTGCAGTT	**T5-64**	**GATAGTGCTACGGTATGTT**	T5-33	GTCGGATTTTAACAGTAAG
T5-10	GAATGGAAGTAATCGCGAT	**T5-80**	**GATAGTGCTACGGTATGTT**	T5-45	GTGACAGAAAACACATACT
**T5-13**	**GAATGTATGCAGTCTCAAA**	T5-43	GCCGACGTCTTAGTTCAAT	T5-46	GTGAGGTTGCATTATGTAT
**T5-1**	**GAATGTATGCAGTCTCAAA**	T5-108	GCGGATCACTTCTTGTTCC	**T5-27**	**GTGATCTATGTATATTGTA**
**T5-23**	**GAATGTATGCAGTCTCAAA**	T5-25	GGACAAAGACAAGTTGTTA	**T5-41**	**GTGATCTATGTATATTGTA**
**T5-35**	**GAATGTATGCAGTCTCAAA**	T5-119	GGGAATTGTATTGAAGCTG	**T5-51**	**GTGATCTATGTATATTGTA**
**T5-40**	**GAATGTATGCAGTCTCAAA**	T5-34	GGGACGGCGTCCACGCTAT	**T5-53**	**GTGATCTATGTATATTGTA**
**T5-65**	**GAATGTATGCAGTCTCAAA**	T5-93	GGGGAGTAGCATATTTAGC	**T5-57**	**GTGATCTATGTATATTGTA**
**T5-68**	**GAATGTATGCAGTCTCAAA**	**T5-7**	**GGGGGAGTGAGTGAATGAT**	**T5-70**	**GTGATCTATGTATATTGTA**
**T5-136**	**GAATGTATGCAGTCTCAAA**	**T5-125**	**GGGGGAGTGAGTGAATGAT**	**T5-89**	**GTGATCTATGTATATTGTA**
T5-110	GAATTGGGGTAAGTATGCT	**T5-12**	**GGGGGAGTGAGTGAATGAT**	**T5-105**	**GTGATCTATGTATATTGTA**
T5-6	GAATTGTCGTATGGACACT	**T5-50**	**GGGGGAGTGAGTGAATGAT**	**T5-137**	**GTGATCTATGTATATTGTA**
T5-19	GACAAGATCTTCATCCCAT	**T5-54**	**GGGGGAGTGAGTGAATGAT**	**T5-131**	**GTGATCTATGTATATTGTA**
T5-11	GACAAGGACTATGTGGGGG	**T5-69**	**GGGGGAGTGAGTGAATGAT**	T5-79	GTGATGATAGGATCAATCT
T5-28	GACAGATGGGAAGTAAGAA	**T5-71**	**GGGGGAGTGAGTGAATGAT**	T5-135	GTGATTCGTCCAAAAGCTT
T5-20	GACAGGAGTTGGAGTGTCT	**T5-75**	**GGGGGAGTGAGTGAATGAT**	T5-107	GTGGACTGAGGTGCTCAGG
T5-31	GACAGTATTGAAGCTGACT	**T5-87**	**GGGGGAGTGAGTGAATGAT**	T5-114	GTGGCGCAGAGTTACATGA
**T5-129**	**GACATTAGTCTAGCAAATT**	**T5-90**	**GGGGGAGTGAGTGAATGAT**	**T5-58**	**GTTAATACGGATTCTCGAG**
**T5-29**	**GACATTAGTCTAGCAAATT**	**T5-106**	**GGGGGAGTGAGTGAATGAT**	**T5-130**	**GTTAATACGGATTCTCGAG**
T5-8	GACCGAAATGTACGACCCC	**T5-139**	**GGGGGAGTGAGTGAATGAT**	T5-113	GTTAGCGCTCTATACTCGT
T5-115	GACGCGCATCGAGGGTAAG	T5-36	GGTATATAGGACGAGAACG	T5-67	GTATGATGATAGATATTGG
T5-77	GACGGACGTTTGTGTTGTA	T5-66	GGTATGACTCAGTCCTCAG	T5-98	GTATGATTGTCTCCTTGTT
T5-91	GACGGACGTTTGTGTTGTG	**T5-138**	**GGTAGAGGTACGCTTAGAA**	T5-32	GTCATGATTGGAAAGCTGT
**T5-14**	**GAGAAAGGCCTGCGTCTCT**	**T5-118**	**GGTAGAGGTACGCTTAGAA**	**T5-59**	**GTTATGTGGACCAAAGCTT**
**T5-30**	**GAGAAAGGCCTGCGTCTCT**	T5-56	GGTGACCTTTCAGCGCAAA	**T5-88**	**GTTATGTGGACCAAAGCTT**
**T5-63**	**GAGAAAGGCCTGCGTCTCT**	T5-38	GGTGACCTTTCAGGGCAAA	T5-95	GTTCGGGTGAAGCACCAGG
T5-2	GAGACAGCGTCATCTCAAA	T5-112	GGTGTGTTAGTGTCTCAAG	T5-3	GTTTATGTCGTATGTGTTC
T5-116	GAGACGGGACACATACGAG	T5-60	GGTTGTATGCAGTCTCAAA		

Bold letters indicate groups of replicate sequence.

**Figure 4:**
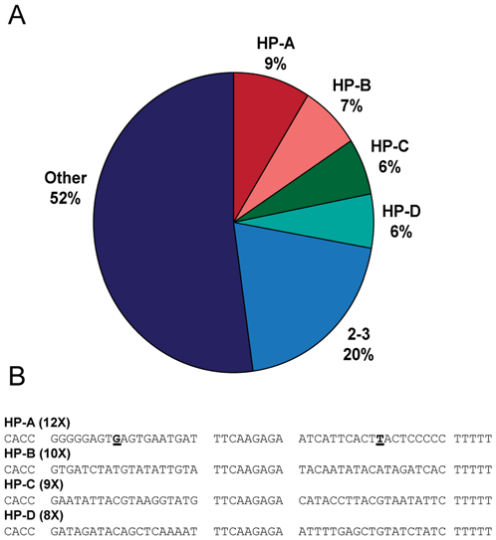
Analysis of sequences of enriched shRNA. (A) Pie chart representing the frequency of shRNA occurrence among the 140 clones sequenced. 2-3X refers to the pool of sequences represented two or three times. Other refers to unique sequences. (B) Sequences of the shRNAs present at the highest frequency. CACC represents the 3′ end of the human U6 promoter. TTCAAGAGA represents the loop sequence. TTTTT represents the termination sequence. Number in parentheses represents the frequency of occurrence out of 140 total sequences. The underlined sequence represents a mismatch within the inverted repeat.

Sequence analysis revealed that approximately half of the total shRNAs sequenced were present at least twice, indicating a significant enrichment as a result of the screening procedure (Fig. 4A). Of particular interest, four shRNA sequences (HP-A, HP-B, HP-C, and HP-D) were present at relatively high frequencies and occurred 12, 10, 9, and 8 times, respectively, in 140 total clones (Fig. 4B). HP-A had a mismatch at position 9 of the sense strand with the antisense strand that corresponded to a G:U wobble which has been shown to have no adverse effects on RNAi activity [35]. Unexpectedly, we were unable to identify by BLAST a single target transcript with perfect complementarity to any of our recovered shRNA sequences.

### Isolated shRNA sequences inhibit MyD88 dependent TNF-α production in macrophages

**Figure 5:**
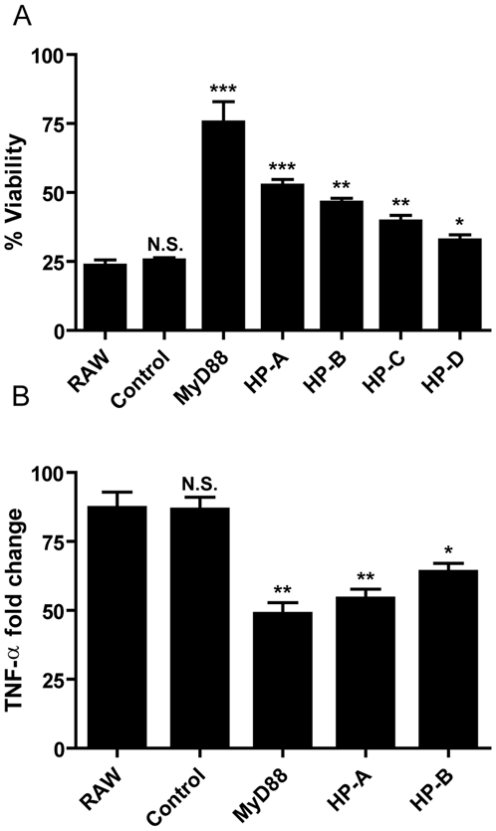
Enriched shRNAs inhibit LPS induced TNF-α gene expression. (A) RAW 264.7 mock infected (RAW), non-specific shRNA (control), MyD88 specific shRNA (MyD88), and enriched shRNA (HP-A and HP-B) infected TNF-Tox cells were stimulated with LPS (2 µg/ml) and doxycycline (1 µg/ml) or just medium alone. Following forty eight hours of stimulation, cell viability was measured using the MTS assay. Percent viability reflects the Abs. 490 of stimulated cells divided by the Abs. 490 of cells treated with medium alone times 100. Data are representative of two independent experiments performed in triplicate. (B) RAW 264.7 mock infected (RAW), non-specific shRNA (control), and MyD88 specific shRNA (MyD88), and enriched shRNA (HP-A and HP-B) infected RAW 264.7 cells were stimulated with LPS (2 µg/ml) or medium alone for 4 hours and analyzed by QPCR. The relative quantities of TNF-α mRNA per sample was calculated against GAPDH using the ΔΔC(T) formula, and normalized as fold change relative to RAW 264.7 incubated with medium alone. N.S. = not significant, *** = p<0.001, ** = p<0.01, * = p<0.05.

To determine if these shRNA sequences targeted genes necessary for the induction of proinflammatory cytokines, we generated lentiviruses containing these hairpin sequences and tested them individually in the TNF-Tox reporter cells. Following LPS stimulation, all four shRNA sequences significantly inhibited LPS-induced DT-A production as determined by increased reporter cell viability (Fig. 5A). Interestingly, the extent of increased viability appeared to correlate with the frequency of the shRNAs in the sequenced pool. As HP-A and HP-B exhibited the most dramatic increase in viability (2.2-fold and 2-fold increase, respectively) over uninfected TNF-Tox cells, we focused on these shRNA sequences for the remainder of our experiments.

To test if the recovered shRNA sequences also inhibited LPS-induced TNF-α gene expression, HP-A and HP-B were transduced into the parental macrophage-like cell line RAW 264.7. Consistent with results from the TNF-Tox reporter cell, both HP-A and HP-B significantly inhibited TNF-α gene expression in RAW 264.7 cells compared to cells infected with a non-specific shRNA sequence (Fig. 5B). The levels of inhibition (38% and 27% for HP-A and HP-B, respectively) approached those seen in cells infected with an shRNA targeting MyD88 (44%), suggesting that both shRNAs targeted a gene (or genes) essential for LPS-induced TNF-α production.

**Figure 6:**
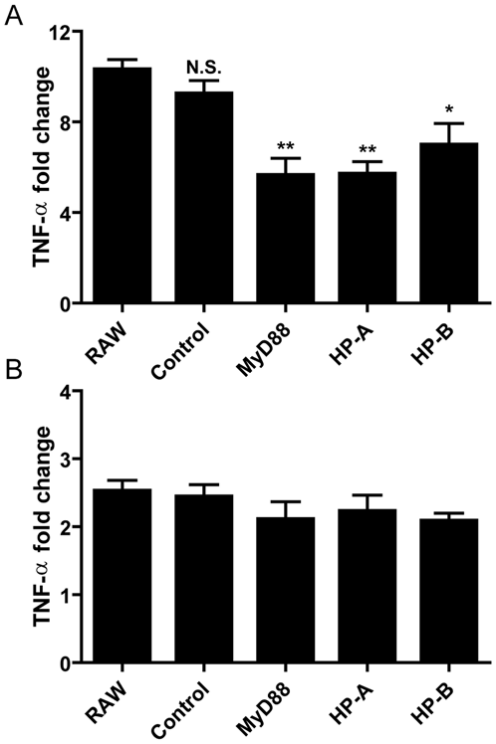
Enriched shRNAs inhibit Pam3Cys but not poly (I:C) dependent TNF-α gene expression. RAW 264.7 mock infected (RAW), non-specific shRNA (control), and MyD88 specific shRNA (MyD88), and enriched shRNA (HP-A and HP-B) infected RAW 264.7 cells were stimulated with (A) Pam3Cys (15 µg/ml), (B) Poly (I:C) (1 µg/ml) or medium alone for 4 hours and analyzed by QPCR. The relative quantities of TNF-α mRNA per sample was calculated against GAPDH using the ΔΔC(T) formula, and normalized as fold change relative to RAW 264.7 incubated with medium alone. N.S. = not significant, ** = p<0.01, * = p<0.05.

TLR4 is unique in that its activation can promote TNF-production through both the MyD88-dependent pathway and the MyD88-independent TRIF pathway [16], [17]. As these signaling pathways are also shared by other TLRs, we tested the ability of our shRNA sequences to inhibit poly (I:C) (TLR3, MyD88 independent) and Pam3Cys (TLR2, MyD88 dependent)-dependent proinflammatory signals. Both HP-A and HP-B inhibited Pam3Cys-dependent TNF-α production by 45% and 32%, respectively (Fig. 6A). In contrast, neither of the two shRNAs inhibited poly (I:C) dependent TNF-α production, suggesting that the target (or targets) of these shRNAs was a component of the MyD88-dependent pathway (Fig. 6B).

## Discussion

Individual TLRs initiate distinct cellular responses. Yet the mechanisms by which these specific signaling cascades are activated remain unclear. The strong association between dysregulation of TLR signaling and the development of chronic inflammatory diseases [20] highlights the need for novel strategies to identify molecular targets that modulate TLR-mediated inflammatory pathways. Using an RNAi-based forward genetic approach, we have identified unique shRNA sequences that inhibit genes necessary for LPS (TLR4) and Pam3Cys (TLR2)-dependent TNF-α production. Importantly, the inhibition of proinflammatory cytokine production by these shRNAs appears to be unique to MyD88-dependent signaling pathways as poly (I:C) (TLR3)-dependent TNF-α production was not inhibited.

One of the hurdles in large-scale gene knockdown experiments is the development of a robust screening system to identify shRNAs with the intended effect. The need for a robust screening system becomes increasingly important as the size of the shRNA library increases, since the majority of shRNAs in a given shRNA library pool will most likely not provide the desired phenotype. Many RNA interference screens rely on the ability of an interfering RNA to rescue a cell from death caused by some cytotoxic drug. However, this approach is very limited as most signaling pathways do not end with cell death. An alternative approach is to use oligonucleotide microarrays to identify the relative enrichment or depletion of individual shRNAs in a large pool (barcode screening) [21], [22]. By comparing the differences in the ratio of shRNA populations in a control population versus a population treated with a selective agent, shRNAs with altered frequencies can be identified. This barcode screening approach can be useful in identifying shRNA targets that are either positively or negatively regulated. However, a major limitation to this system is that the selective agent must provide a specific growth advantage or disadvantage.

In the present study, we have developed a simple positive selection approach for identifying essential components of signaling pathways, such as TLR signaling, where ligand binding does not alter cell viability. By placing DT-A production under the control of the TNF-α promoter, we were able to positively select shRNA sequences which inhibited LPS induced TNF-α production. Positive selection results in the complexity of the shRNA library pool decreasing significantly after each round of selection, thereby increasing the likelihood of identifying shRNA sequences that inhibit the targeted pathway. Importantly, this DT-A reporter system can be modified for use with other promoter systems and should greatly facilitate the identification of participants of various signaling pathways.

RNAi was initially considered to be a sequence-specific gene silencing mechanism that required a perfect match between the guide strand of the siRNA and the mRNA target sequence [36]. Under this assumption, much effort has been expended attempting to use RNAi to knockdown expression of specific genes. However, more recently, the issue of specificity has been questioned as several groups have demonstrated that various mismatches between the guide strand of the siRNA and the mRNA target do not abolish the silencing effect of the siRNA [37], [38]. Additionally, microarray analyses have demonstrated that siRNAs with only partial complementarity to the 3′ untranslated region of mRNAs can cause reduction in the RNA levels of a large number of transcripts, a phenomenon termed “off-target” effects [39], [40]. Such “off-target” effects greatly increase the difficulty of identifying the genes actually responsible for the selected phenotype.

Although verification of the siRNA target gene can be easily done by generating multiple siRNAs targeting other regions of the gene, this strategy becomes problematic if the phenotype caused by the siRNA is mediated strictly by an “off-target” effect. A recent example of this is a study by Lin et al. in which all of the top candidate siRNAs from their siRNA screen were found to elicit off-target gene silencing[41]. Through the use of gene expression profiling and bioinformatic analysis, Lin et al. were able to identify Mcl-1 as the “off-target” gene responsible for resistance to the small molecule ABT-737 in their siRNA screen. Interestingly, the library used in their study contained several siRNAs that were designed to target Mcl-1. However, none of the four Mcl-1-specific siRNAs from their library were able to sensitize cancer cells to ABT-737 induced cytotoxicity, whereas the top candidate siRNAs from their screen, specific for FGFR2, TNFRSF13B, and PRDM13, were able to inhibit Mcl-1 gene expression through partial complementarity of the siRNAs to the 3′ untranslated region of Mcl-1. Thus, caution needs to be taken when interpreting data obtained from RNAi libraries as specific siRNAs do not always target their intended gene and in some cases may exhibit “off-target” effects.

Recently, it has been shown that overexpression of Argonaute-2 (Ago2) can enhance the specificity and potency of shRNAs for mRNA targets with perfectly matched binding sites [42]. Thus, the ectopic expression of Ago2 in our TNF-Tox cell line may increase the likelihood of identifying perfectly matched shRNA sequences. However, it should be noted that in the case of Lin et al., identification of Mcl-1 as a target for sensitizing cancer cells to ABT-737 would not have been possible if siRNAs with partial complementarity to Mcl-1 were not present to downregulate Mcl-1 through “off-target” effects. Thus, shRNA libraries with greater diversity, and potentially greater “off-target” gene silencing, such as our random shRNA library, may provide greater genome wide coverage and increase the probability of identifying small RNAs capable of modulating complex cellular signaling pathways in a phenotype driven screen. Although the bona fide targets of our shRNA sequences could not be identified using target identification algorithms such as BLAST, the recovered shRNA sequences were confirmed to be potent inhibitors of MyD88-dependent inflammation and may have therapeutic implications for the treatment of chronic inflammatory disorders.

In summary, we have developed a completely random shRNA library and a novel reporter cell line for large-scale genomic screens. Importantly, any RNA species could potentially be targeted using this shRNA library (for example, noncoding RNA transcripts, alternatively spliced transcripts, or viral RNAs) in virtually any mammalian cell. The use of such random shRNA libraries for gene discovery coupled with stringent selection systems can facilitate the rapid identification of small RNA modulators involved in various biological processes.

## Materials and Methods

### Design of oligonucleotide for generation of random shRNA library

An oligonucleotide containing 20 base pairs of the 3′ end of the U6 promoter (5′-GGAAAGGACGAAACACC-3′) followed by a “G,” the transcriptional start point of the human U6 promoter, 18 randomized oligonucleotides, a loop sequence (5′-TTCAAGAGA-3′) [32], and extra sequences that form a hairpin structure that contains a 32 base pair inverted repeat sequence with a TTCG loop sequence (underlined) (5′-TTCAAGAGATGCATAGCT CCACATACTCCAGAATGACTTAATTCGTTAAGTCATTGTGGAGTATCTGGAGCTATGCATCTCTTGAA-3′) was purchased from Integrated DNA Technologies (Coralville, IA) (Fig. 1A).

### Generation of random shRNA library

The procedure for the production of the shRNA library is shown schematically in Figure 1B.

### Step 1

300 picomoles of the 120 base pair oligonucleotide was denatured at 95°C for 10 minutes in 50 µl of NEB #2 restriction enzyme buffer (New England Biolabs, Ipswitch, MA) and slowly cooled at a rate of −1°C/min to allow formation of the hairpin structure of the oligonucleotide. Three nanomoles of U6 blocker primer (5′-GGTGTTTCGTCCTTTCC-3′) complementary to the U6 sequence of the long oligonucleotide was also added to the mixture to stop transcription by T4 DNA polymerase in the following step.

### Step 2

The gap between the 3′ end of the oligonucleotide and the 5′ end of the U6 blocker was filled using T4 DNA polymerase (New England Biolabs), which does not have strand displacement activity, at 37°C for 30 minutes in the presence of 2 mM dNTP.

### Step 3

To remove the short U6 blocker and purify the gap-filled oligonucleotide, the reaction mixture from step B was resuspended in 1x PAGE loading buffer (8 M urea, 0.04% bromophenol blue (BPB), 0.04% xylene cyanol FF, 40 mM Tris pH 8.0, 0.001 mM EDTA) and subjected to denaturating Urea PAGE. The DNA band of the gap-filled oligonucleotide was excised from the gel and purified.

### Step 4

A poly-thymidine tail was added at the end of the gap-filled oligonucleotide using terminal transferase (NEB) in the presence of 1 uM dTTP according to the manufacturer's instruction. This poly-T tail functions as a termination sequence for RNA polymerase.

### Step 5

The resulting oligonucleotide was purified using urea polyacrylamide gel electrophoresis and used as a template to generate double stranded DNA using exonuclease deficient Klenow fragment (New England Biolabs) and the primer, dsHP (AGCUATAGTTTAGCGGCCGCTTATACTACTCAAAAAAAAAAAAAAAAAA) according the manufacturer's instructions. Following agarose gel electrophoresis the PCR fragment was gel purified using QIAquick gel extraction kit (Qiagen, Valencia, CA) and resuspended in 60 µl of water for use in the subsequent PCR amplification.

### Step 6

Next the DNA was amplified using 1 µl of DNA template from step 5, 3 mM dNTP, 3 mM MgCl_2_, 1X PCR buffer, and 3 picomoles of the uracil containing primers, HP-up primer (5′-Biotin-TTTCGA/deoxyU/TTCTTG GCTTTATATATCTTGTGGAAAGGACGAAACACC-3′) and HP-lo primer (5′-Biotin-GCC/deoxyU/ATAGTTTAGCGGCCGCTTATACTACTCAAAAAAAAAAAA AAA-3′) mixed with 2.5 units of Taq DNA polymerase (Invitrogen, Carlsbad, CA). PCR was performed under the following conditions: 2 minutes at 95°C, 30 seconds at 50°C, and 30 seconds at 72°C. To avoid formation of nonspecific bands, the optimal number of PCR cycles was determined empirically.

### Step 7

To generate cohesive ends, the amplified PCR product was treated with the USER enzyme (New England Biolabs) according to the manufacturer's instructions, which specifically removes deoxyuridine in the DNA. The final product contains unique 7 bp and 4 bp overhangs at each end, which were used to facilitate cloning into the lentiviral vector pLL3.7 (kindly provided by Dr. Luk Van Parijs).

### Step 8

Finally, samples were digested with BpmI to remove the extra sequences between the inverted repeat sequences, blunt-ended and religated to generate the final product which contained the following: the human U6 promoter including the “G” transcription start site, a random 18-mer sequence, a TTCAAGAGA loop sequence, the 19-mer sequences complementary to the random sequence, followed by a poly-T termination sequence.

### Cells and reagents

RAW 264.7 is a murine macrophage cell line, kindly provided by Dr. Genhong Cheng (University of California Los Angeles, Los Angeles, CA). RAW 264.7 cells were grown in Dulbecco's modified Eagle medium (DMEM) supplemented with 10% calf serum, 2 mM L-glutamine, 100 U/ml penicillin, and 100 µg/ml streptomycin. Escherichia coli LPS (serotype O55:B5) and doxycycline were obtained from Sigma (St. Louis, MO), polyinosinic:polycytidylic acid (poly (I:C)) was obtained from Amersham Biosciences (Piscataway, NJ) and Pam3Cys was obtained from Alexis Biochemicals (San Diego, CA). The following concentrations were used for stimulation: poly (I:C) (TLR3, 1 µg/ml), LPS (TLR4, 2 µg/ml), and Pam3Cys (TLR2, 15 µg/ml).

### Plasmid constructs for reporter cell

The murine TNF-α promoter fragment was isolated from the plasmid TNF-Luc [43], kindly provided by Dr. Peter Johnson (National Cancer Institute, Frederick, MD), by digestion with BamHI, blunt-ended by treatment with DNA polymerase I (Klenow), and subsequently digested with HindIII. The resulting TNF-α promoter fragment was inserted into the plasmid pcDNA3.1 (Invitrogen) that had been digested with AccI, blunt-ended by treatment with DNA polymerase I (Klenow), and subsequently digested with HindIII to remove the CMV prompter. The coding sequence of the wild-type diphtheria toxin A (DT-A) catalytic fragment, the attenuated DT-A (Tox-176) with a G128D mutation [34], or enhanced (E)GFP was isolated from the plasmids pBK-CMV-DTA, pBK-CMV-Tox176 [44] [both kindly provided by Dr. Ronald Rodriguez (Johns Hopkins University, Baltimore, MD], or pEGFP-N1 (Clontech, Mountain View, CA), respectively, and cloned downstream of the TNF-α promoter to generate the reporter plasmids TNF-DTA, TNF-Tox176, and TNF-EGFP. A Tet-On expression system was used to decrease the basal levels of TNF-promoter induced gene expression. Two TetO_2_ sites were inserted by PCR into each of the three reporter plasmids 10 bp downstream of the TNF-promoter TATA box to generate TNF-TetO_2_-DTA, TNF-TetO_2_-Tox176, and TNF-TetO_2_-EGFP.

### Generation of macrophage reporter cells

RAW 264.7 cells (2.5×10^6^ cells) were plated overnight in a 60 mm dish. The following day, the cells were transfected with 16.5 µg of reporter plasmid using Superfect reagent (Qiagen) according to the manufacturer's instructions. Two days later, the transfected cells were seeded in 96-well plates and selected for stable expression by 2.5 mM histidinol (Sigma). Histidinol resistant cells were cloned by limiting dilution and stimulated with LPS (1 µg/ml) to assay for DT-A induced cytotoxicity or EGFP fluorescence, as measured by CellTiter 96 AQueous (MTS) solution (Promega, Madison, WI) or flow cytometry, respectively.

The generation of the tetracycline-inducible reporter plasmids required two rounds of stable transfection. First, RAW 264.7 cells were transfected with pcDNA6/TR (Invitrogen) using Superfect transfection reagent, as above, and selected for stable expression by G418 (1 mg/ml). Antibiotic resistant clones were assayed for Tet-repressor expression by transfecting individual clones with TNF-TetO_2_-EGFP and monitoring EGFP fluorescence by flow cytometry. Clones with decreased EGFP fluorescence compared to parental RAW 264.7 transfected cells were pooled and stably transfected with the individual Tet-inducible reporter plasmids. Histidinol (2.5 mM) resistant cells were cloned by limiting dilution and assayed for DT-A induced cytotoxicity or EGFP fluorescence as above in the presence or absence of doxycycline (1 µg/ml).

### Generation of control shRNA-expressing lentiviral vectors

The viral backbone of our lentiviral expression constructs is pLL3.7. PCR was performed to generate an shRNA expression cassette containing the human U6 promoter, the specific shRNA target sequence (containing the 19-mer sense sequence, the TTCAAGAGA loop sequence, followed by the 19-mer antisense sequence), followed by a poly-T termination sequence flanked by the restriction sites XbaI and XhoI. The PCR fragment was digested with XbaI and XhoI, and then inserted into the unmodified pLL3.7. Primers 5′-ATCGATTCTAGAAAGGTCGGGCAGGAAGAGGG-3′ and 5′-ATCGATCTCGAGAAAAATTCTCCGAACGTGTCACGTCTCTTGAACGTGACACGTTCGGAGAACGGTGTTTCGTCCTTTCCACAAG-3′ were used to generate a scrambled non-specific Control shRNA. Primers 5′-ATCGATTCTAGAAAGGTCGGGCAGGAAGAGGG-3′ and 5′-GATCTCGAGAAAAAGCCAGCGAGCTAATTGAGAAACTCGAGTTTCTCAATTAGCTCGCTGGCCCGGTGTTTCGTCCTTTCCACAAG-3′ were used to generate the MyD88 specific shRNA.

### Virus production

Lentiviruses were produced by cotransfecting HEK-293T cells with the shRNA library lentivirus expression plasmid, the HIV-1 lentiviral packaging constructs pRSV-Rev and pMDLg/pRRE, and the VSV-G expression plasmid pMD2G, kindly provided by Dr. Didier Trono (University of Geneva Medical School, Geneva, Switzerland), using the calcium phosphate transfection method. Lentiviral supernatant was harvested at 48 and 72 hours post-transfection, passed through a 0.45-µm filter, ultracentrifuged for 90 minutes at 19,400 rpm in a SW28 rotor, resuspended in 200 µl of 10% CS in DMEM, and stored at −80°C until use. The infectious titer was approximately 10^7^ and 10^8^ transducing units/ml on RAW 264.7 cells and 293T cells, respectively, as determined by flow cytometry.

### Large-scale lentiviral shRNA screen

TNF-Tox reporter cells (1.5×10^7^ cells/dish) were seeded overnight in 150 mm plates and transduced the following day with sufficient lentiviral particles from our shRNA library to infect 80% of the cells as determined by flow cytometry. Following a 72 hour incubation, transduced cells were harvested, re-seeded (1.5×10^7^ cells/dish) in 150 mm plates, and treated overnight with doxycycline (1 µg/ml). The following day, cells were washed and stimulated with 1 µg/ml LPS in the presence of doxycycline (1 µg/ml) and incubated for 5–8 days to allow expansion of surviving clones.

### Recovery of shRNA sequences and reinfection

Genomic DNA from surviving cells was isolated using Qiagen DNeasy kit (Qiagen). PCR amplification of the shRNA inserts was performed with Platinum Taq DNA polymerase (Invitrogen) using the following primers: 5′-ATCGATTCTAGAAAGGTCGGGCAGGAAGAGGG-3′ and 5′-ATGCATGGCGGTAATACGGTTATCC-3′. The PCR fragment was digested with XbaI and XhoI, and then inserted into the unmodified pLL3.7 lentivirus vector. Generation of lentivirus and screening was performed as described above. After five rounds of enrichment, PCR amplified shRNA inserts were subcloned into the pLL3.7 lentivirus vector and individual clones sequenced to determine the shRNA sequence.

### RNA isolation and quantitative PCR (QPCR) analysis

RNA was isolated from RAW 264.7 cells using TRIzol Reagent (Invitrogen), and cDNA was synthesized using the SuperScript III First-Strand Synthesis SuperMix (Invitrogen). SYBR Green reactions were conducted with the IQ SYBR Green mix (Bio-Rad, Hercules, CA). Reactions were run on the MJR Opticon Continuous Fluorescence detector (Bio-Rad) and analyzed with Opticon Monitor Software 1.08 (Bio-Rad). The relative quantities of the gene tested per sample were calculated against GAPDH using the ΔΔC(T) formula. The following primers were used: TNF-up – 5′-ATGAGCACAGAAAGCATGATC-3′; TNF-down – 5′-TACAGGCTTGTCACTCGAATT-3′; GAPDH-up – 5′ GTTGCCATCAATGACCCCTTCATTG-3′; GAPDH-down – 5′-GCTTCACCACCTTCTTGATGTCATC-3′.
